# Inhibition of phosphodiesterase 5 restores endothelial function in renovascular hypertension

**DOI:** 10.1186/s12967-014-0250-x

**Published:** 2014-09-16

**Authors:** Ananda T Dias, Amanda S Cintra, Jéssica C Frossard, Zaira Palomino, Dulce E Casarini, Isabele BS Gomes, Camille M Balarini, Agata L Gava, Bianca P Campagnaro, Thiago MC Pereira, Silvana S Meyrelles, Elisardo C Vasquez

**Affiliations:** Laboratory of Translational Physiology, Health Sciences Center, Federal University of Espirito Santo, Vitoria, ES Brazil; Emescam School of Health Sciences, Vitoria, ES Brazil; Department of Medicine, Nephrology Division, Federal University of Sao Paulo, Sao Paulo, SP Brazil; Pharmaceutical Sciences Graduate Program, Health Sciences Center, Federal University of Espirito Santo, Vitoria, ES Brazil; Department of Physiology and Pathology, Health Sciences Center, Federal University of Paraiba, João Pessoa, PB Brazil; Pharmaceutical Sciences Graduate Program, University Vila Velha (UVV), Vila Velha, ES Brazil; Federal Institute of Education, Science and Technology (IFES), Vila Velha, ES Brazil

**Keywords:** Sildenafil, Oxidative stress, Renovascular hypertension, Angiotensin, Endothelial function, 2K1C

## Abstract

**Background:**

The clipping of an artery supplying one of the two kidneys (2K1C) activates the renin-angiotensin (Ang) system (RAS), resulting in hypertension and endothelial dysfunction. Recently, we demonstrated the intrarenal beneficial effects of sildenafil on the high levels of Ang II and reactive oxygen species (ROS) and on high blood pressure (BP) in 2K1C mice. Thus, in the present study, we tested the hypothesis that sildenafil improves endothelial function in hypertensive 2K1C mice by improving the NO/ROS balance.

**Methods:**

2K1C hypertension was induced in C57BL/6 mice. Two weeks later, they were treated with sildenafil (40 mg/kg/day, via oral) or vehicle for 2 weeks and compared with sham mice. At the end of the treatment, the levels of plasma and intrarenal Ang peptides were measured. Endothelial function and ROS production were assessed in mesenteric arterial bed (MAB).

**Results:**

The 2K1C mice exhibited normal plasma levels of Ang I, II and 1–7, whereas the intrarenal Ang I and II were increased (~35% and ~140%) compared with the Sham mice. Sildenafil normalized the intrarenal Ang I and II and increased the plasma (~45%) and intrarenal (+15%) Ang 1–7. The 2K1C mice exhibited endothelial dysfunction, primarily due to increased ROS and decreased NO productions by endothelial cells, which were ameliorated by treatment with sildenafil.

**Conclusion:**

These data suggest that the effects of sildenafil on endothelial dysfunction in 2K1C mice may be due to interaction with RAS and restoring NO/ROS balance in the endothelial cells from MAB. Thus, sildenafil is a promising candidate drug for the treatment of hypertension accompanied by endothelial dysfunction and kidney disease.

## Introduction

High blood pressure (BP) has become a health issue, with a projected prevalence of about one third of the global population worldwide by 2025 [1], and endothelial dysfunction is the hallmark of this condition [2]. Among the different types of high BP, renovascular hypertension is an important cause of secondary hypertension, which is characterized by increased activation of the renin-angiotensin system (RAS) [3-5]. The prevalence of renovascular hypertension has been estimated to be 5% of all hypertensive individuals, but it can reach higher percentage depending on the population’s characteristics, e.g., renal artery stenosis can reach approximately 5% of persons aged ≥ 65 years [6,7]. We have focused this study on this secondary hypertension because these patients are highly susceptible to develop resistance to conventional antihypertensive drugs, increasing the morbidity and mortality [4,8]. Thus, there is a need of studies to a better understanding and to develop new therapeutic approaches against the progression of this disease.

Experimental renovascular hypertension was introduced by Goldblatt in 1934 in dogs by performing unilateral clipping of the renal artery, referred to as the two-kidney, one-clip (2K1C) model. Three decades later, the rat became the main experimental model of 2K1C hypertension in our and other laboratories [9-11]. However, genetic discoveries and advances in molecular biotechnologies have provided the opportunity to develop mouse models of cardiovascular diseases, which has led to an increasing number of studies using this murine model of hypertension [12-15]. Physiologically, it is well known that unilateral renal artery stenosis reduces renal perfusion, which activates the RAS [5]. Recently, this system has been extended by the addition of a novel axis consisting of the angiotensin-converting enzyme 2 (ACE2), the heptapeptide angiotensin (Ang) 1–7, and the G protein-coupled receptor Mas [16]. ACE2 converts the vasoconstrictive and pro-oxidative peptide Ang II to Ang 1–7, which exerts vasodilatory and antioxidant effects via its receptor, Mas [16-19]. Although our laboratory analyzed some RAS peptides during the development phase of 2K1C hypertension in a murine model [3,20,21], in the present study, we extended the analysis of the Ang I, II and 1–7 levels in both plasma and the stenotic kidney during the established phase of renovascular hypertension.

Endothelial dysfunction is commonly observed in chronic arterial hypertension and is characterized by impaired nitric oxide (NO) bioavailability and increased reactive oxygen species (ROS) levels. In recent years, our laboratory [15,22,23] and others [24,25] have investigated microvascular endothelial dysfunction in various models of cardiovascular disease, including the 2K1C mouse model of hypertension, which has greatly contributed to our understanding of the relationship between endogenous activation of the RAS and endothelial dysfunction in resistance arteries [15]. This model provides suitable approach to evaluate the efficacy of new drugs for the treatment of renovascular hypertension.

The phosphodiesterase 5 (PDE5) inhibitor sildenafil, which increases the vascular signaling of the NO/cGMP pathway [26,27], has successfully been used to treat erectile dysfunction and pulmonary hypertension [28], emerging as a promising alternative therapy for systemic cardiovascular dysfunctions. Our laboratory has recently shown that sildenafil was able to restore the endothelial dysfunction and blood mononuclear and liver cell DNA damage, which accompany atherosclerosis [27,29]. Moreover, Dias et al. [21] recently reported that sildenafil ameliorates the oxidative damage to the stenotic kidney and reduces the intrarenal levels of Ang II in the 2K1C mouse.

Therefore, the present study was designed to evaluate the effect of chronic treatment with sildenafil on high BP, the levels of peptide components of the RAS and vascular function in the hypertensive 2K1C mouse. We hypothesized that sildenafil reduces the deleterious effects of Ang II, increases the levels of the counterbalancing peptide Ang 1–7 and restores the endothelial function of resistance vessels in hypertensive 2K1C mice. The confirmation of this hypothesis may provide a novel pharmacological approach to treat endothelial dysfunction and, especially, resistant hypertension.

## Materials and methods

### Animals

Experiments were performed in male wild-type mice (C57BL/6) that weighed 23 g on average (10-week-old). Mice were bred and maintained in the Laboratory of Translational Physiology animal facility (Vitoria, ES, Brazil) and were fed with a standard chow diet and received water *ad libitum*. Animals were housed in individual plastic cages with automatic controlled temperature (22°C) and humidity (60%) and were exposed to a 12/12 h light–dark cycle. All of the experimental procedures were performed in accordance with the National Institutes of Health (NIH) guidelines, and the study protocols were approved by the Institutional Animal Care and Use Committee (CEUA-EMESCAM Protocol # 02/2013).

### Induction of 2K1C renovascular hypertension and treatment

The 2K1C Ang-dependent hypertension was induced as previously described [3,12,15,21,30]. Briefly, animals were anesthetized (91/9.1 mg/kg ketamine/xylazine, i.p.). The left renal artery was exposed through a retroperitoneal flank incision and carefully isolated from the renal vein, nerves, and connective tissues. Using an ophthalmic surgical microscope (Opto Eletronica SA, model SM 2002, Belo Horizonte, MG, Brazil), a U-shaped stainless steel clip with a 0.12 mm wide opening was placed around the renal artery near the abdominal aorta, which decreased renal perfusion [31]. Two weeks after surgery, animals were divided into two groups (8 to 10 animals per group): renovascular hypertensive mice treated with vehicle (2K1C) and hypertensive mice treated with 40 mg/kg/day of the PDE5-inhibitor sildenafil (Viagra®, Pfizer) for 2 weeks by oral gavage (2K1C-sildenafil). Sham-operated mice were used as a normotensive control group. The effectiveness of this sildenafil dose [32] was previously demonstrated in studies on endothelial dysfunction and DNA damage in our laboratory [21,27,29]. The outcomes were examined 28 days after induction of hypertension.

### Hemodynamic measurements

Twenty six days after 2K1C procedure or sham operation, mice were anesthetized with a combination of ketamine/xylazine (91/9.1 mg/kg, i.p.) and a catheter (0.040 mm outer × 0.025 mm inner diameters, MicroRenathane, Braintree Science, Massachusetts, USA) was inserted into the right carotid artery for the measurement of mean arterial pressure (MAP) and heart rate (HR) recordings. The free catheter end was tunneled under the skin of the back to the level of the shoulder blades. Hemodynamic measurements were performed in conscious, freely moving mice in their own cages, two days after catheter placement, as already validated as a sufficient period for complete recovery from surgery by others [33] and standardized in our laboratory [3,13-15]. For the MAP and HR recordings, the arterial catheter was plugged into a disposable BP transducer (Cobe Laboratories, Colorado, USA) connected to a pressure processor amplifier and data-acquisition system (MP100, Biopac Systems, California, USA). At the beginning of the experimental session, a period of approximately 30 min was allowed for stabilization of cardiovascular parameters before the measurement of basal MAP and HR values in conscious mice (Acknowledge software, Biopac Systems).

### Assessment of endothelial function

At the end of the treatment, animals were anesthetized using sodium pentobarbital (50 mg/kg, i.p.), and the mesenteric arterial bed (MAB) was isolated and prepared for vascular studies. The superior mesenteric artery was cannulated. Then, the MAB was transferred to a 37°C chamber and was perfused using a peristaltic pump (Peri-Star Pro 4-channel pump, WPI, Lu Jia Zui District, Shangai, China) at a constant flow rate (3 mL/min) with oxygenated (95% O_2_ - 5% CO_2_ mixture) physiological salt solution (in mmol/L: 130 NaCl, 4.7 KCl, 1.6 CaCl_2_•2H_2_O, 1.18 KH_2_PO_4_, 4.7 MgSO_4_•7H_2_O, 14.9 NaHCO_3_, 0.026 EDTA, 11.1 glucose, pH 7.4). The perfusion pressure was monitored using a T-tube inserted between the pump and the inflow cannula that was connected to a pressure transducer and a data acquisition system (Biopac Systems). Then, dose–response curves of acetylcholine (ACh, 3 × 10^−7^ to 3 × 10^−2^ M) and sodium nitroprusside (SNP, 3 × 10^−7^ to 3 × 10^−2^ M) were generated in the isolated MABs. The vascular responses were evaluated based on the changes in the perfusion pressure and the vasodilator responses to ACh and SNP, which were calculated as a percentage of the pre-contraction induced by norepinephrine (NE, 9.8 × 10^−6^ M). To assess the mechanisms underlying the vascular effect of ACh dose–response curves were generated in separate preparations containing an intact endothelium and using specific blocker agents: (a) the nonselective NO synthase (NOS) inhibitor N-nitro-L-arginine methyl ester (L-NAME, 10^−4^ M), (b) the nonselective inhibitor of cyclooxygenases 1 and 2 (Cox1 and Cox2) indomethacin (10^−5^ M) or (c) the NAD(P)H oxidase inhibitor apocynin (30 μM). The relaxation responses to ACh were expressed as the percentage of dilation relative to the maximal pre-contraction level. For each curve, the maximum effect (R_max_) and the log of the concentration of the agonist that produced half of R_max_ (log EC_50_) were calculated via nonlinear regression analysis. The sensitivities of the agonists were expressed as pEC_50_ (−log EC_50_). The difference in the area under the curve (Δ AUC) for each of the responses of the MAB to ACh in the presence of each inhibitor were calculated, and these results were expressed in arbitrary units (a.u.).

### Measurements of the angiotensin peptide levels

The plasma and intrarenal levels of Ang I, II and 1–7 were analyzed via high performance liquid chromatography (HPLC), as previously reported [20,21,30]. For this analysis, blood was collected in the presence of EDTA and a protease inhibitor cocktail (Product # P2714, Sigma-Aldrich), and after centrifugation (9.5 *g*, for 20 min) using a refrigerated centrifuge (4°C), plasma was collected for further analysis. The Ang peptides were extracted using Oasis C18 columns previously activated with methanol (5 mL), tetrahydrofuran (5 mL), hexane (5 mL) and water (10 mL). After activation, the samples were eluted in ethanol/acetic acid/water at proportions 90% - 4% - 6%. The eluates were dried, re-dissolved in 500 μL of mobile phase A (5% acetonitrile in 0.1% phosphoric acid) and filtered for analysis by HPLC. The Ang peptides in each sample were separated using a reverse-phase ODS Aquapore 300 (250 × 4.6 mm) HPLC column with a particle size of 7 μm (Perkin-Elmer’s Brownlee Columns, Norwalk, USA) using a 5–35% gradient of mobile phase B: 95% acetonitrile in 0.1% phosphoric acid at a flow of 1.5 mL/min for 40 min. The Ang peptides were identified by comparing them with the retention times and peak heights of standard peptides.

For renal analysis of the Ang peptides, they were extracted from kidney homogenates and purified as performed on plasma samples. The subsequent steps, from the activation step to the HPLC analysis step using the reverse-phase column, the procedures were the same as those for plasma sample analysis. The Ang peptides were identified based on the retention time (<6%) and peak height (<5%) of standard Ang peptides and were normalized according to the kidney weight.

### Isolation and identification of endothelial cells from MAB

At the end of the treatment, animals were anesthetized with sodium pentobarbital (50 mg/kg, i.p.), the midline of the abdomen was incised and the MAB was isolated, minced and digested with type II collagenase (1000 U/mL) at 37°C for 60 min at constant agitation. The cellular digest was filtered through a sterile 70-μm nylon mesh to remove cell debris, centrifuged at 400 *g* for 10 min, and washed twice in PBS. The cell pellet was resuspended in freezing solution and stored at −80°C for further analysis. For flow cytometry analysis, the samples were thawed using a heated orbital shaker at 37°C and immediately transferred to a round-bottom tube, in which DMEM containing 20% FBS was added in a drop wise fashion during a gentle agitation. The presence of endothelial cells in MAB digest was confirmed using an APC-conjugated monoclonal antibody against APCCAM-1 (CD31-PE). After thawed, MAB digest cell samples were resuspended at a concentration of 1 × 10^5^ cells/mL in PBS and incubated with 5 μL of CD31-APC for 20 min in the dark and at room temperature. In the flow cytometry analysis, a APC-conjugated rat IgG_2a_ was used as an isotype-specific control to set the threshold values. From each sample, 100,000 events have been generated by a FACSCanto II flow cytometer (Becton Dickinson - BD, San Juan, CA, USA) using an appropriated filter for APC (660 nm).

### Measurement of endothelium ROS

The ROS analysis was performed by flow cytometry using dihydroethidium (DHE), diaminofluorescein (DAF) and hydroxyphenyl fluorescein (HPF) to detect intracellular •O_2_^−^, NO and •ONOO^−^, respectively, as previously described [21,31]. Briefly, 160 mM of DHE or 10 μM of HPF or 2 μM of DAF was added to the cell suspension (10^6^ cells) and incubated at 37°C for 30 min (DHE and HPF) or 180 min (DAF) in the dark. For positive control, samples were treated for 5 min with 50 mM H_2_O_2_ and/or 100 μM of SNP to create oxidative stress without being toxic to the cells, whereas for negative control, the cells were incubated with ethanol. Cells were then washed, resuspended in PBS and kept on ice for immediate detection by flow cytometry (BD). For quantification of DHE, DAF and HPF fluorescence, 100,000 events were acquired and data were analyzed using the FACSDiva software (BD).

### Statistical analysis

The values are expressed as the means ± S.E.M. The Kolmogorov-Smirnov test indicated that the variables displayed a normal (Gaussian) distribution. Statistical comparisons between more than two means were performed using one-way or two-way analysis of variance (ANOVA) followed by Bonferroni’s *post hoc* test. The statistical analyses were performed using Prism software (Prism 6, GraphPad Software, Inc., San Diego, CA, USA). A value of p < 0.05 was considered to be statistically significant.

## Results

### Body and kidney weights

The initial body weight was similar between the groups. By the end of the experiments, only the non-treated 2K1C group displayed reduced body weight (−7%) compared with the Sham group (26±0.5 g) and the 2K1C group treated with sildenafil (25±0.3 g). Twenty-eight days after surgery, the left clipped kidney atrophied (29±2.6 mg, p < 0.05), whereas the right non-clipped kidney displayed compensatory hypertrophy (49±0.3 mg, p < 0.05) in the non-treated 2K1C mice compared with the Sham mice (42±2.0 and 45±1.2 mg, respectively). As previously reported by our laboratory [21], sildenafil not only reduced the renal atrophy of the clipped kidney (38±0.7 mg, p < 0.05) but also attenuated the compensatory hypertrophy of the contralateral kidney (43±1.2 mg, p < 0.05).

### Blood pressure and heart rate

**Figure 1:**
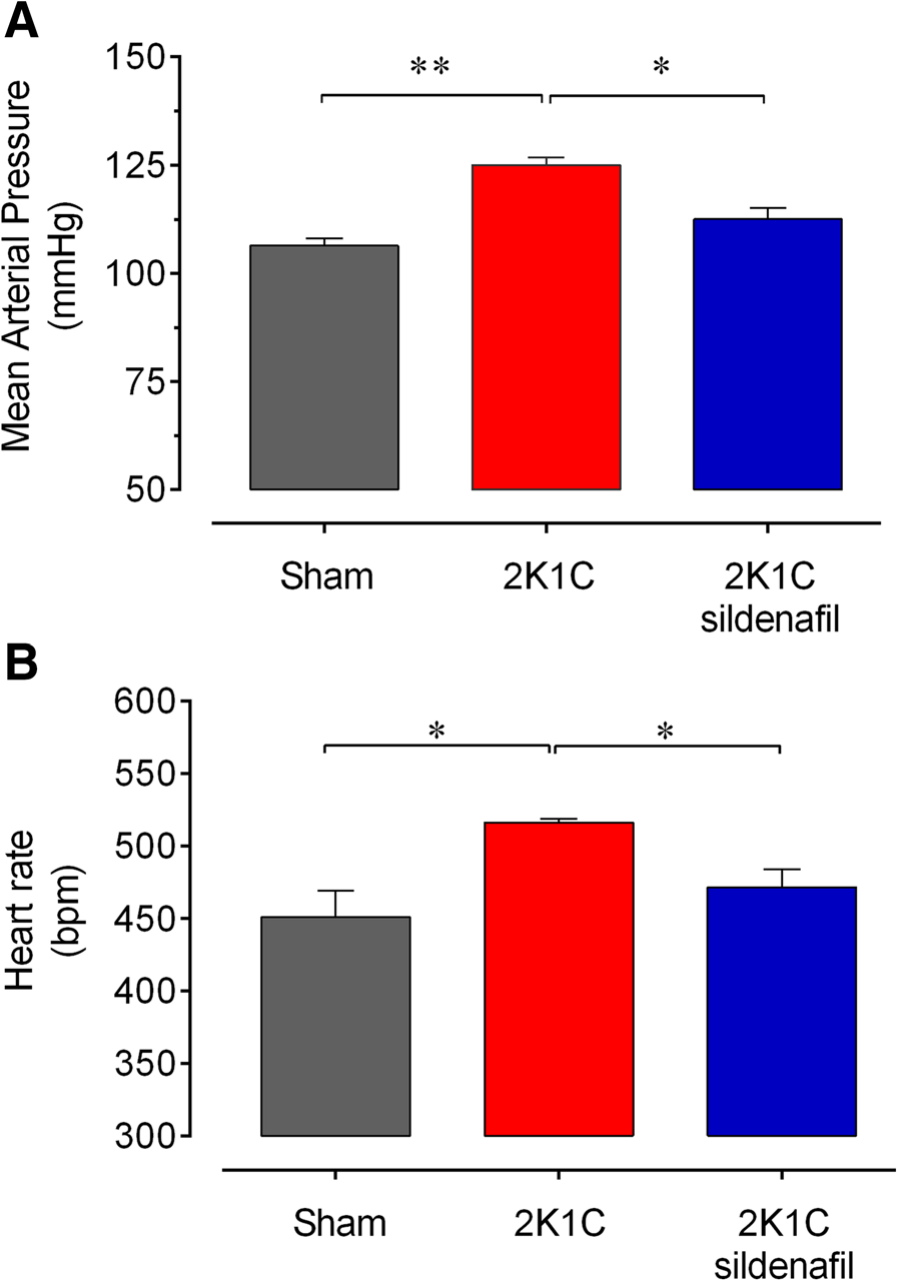
**Effects of sildenafil on hemodynamics.** Resting arterial blood pressure **(A)** and heart rate **(B)** in 2K1C hypertensive mice four weeks after renal artery clipping compared with non-treated mice 2K1C and Sham mice. Values are means±SEM for n = 6 to 8 animals per group. *p < 0.05 and **p < 0.01, one-way ANOVA.

The average values of resting BP and HR measured in conscious animals 28 days after renal artery clipping are summarized in Figure 1. As expected, the 2K1C mice exhibited a higher BP (26%, p < 0.01) than the sham mice (104±2 mmHg), and sildenafil treatment reduced these levels to values similar to those of the Sham mice (Figure 1A). The resting HR of the 2K1C mice was significantly higher (+14%, p < 0.05) than that of the Sham mice (Sham: 451±18 bpm), but no significant difference was detected after sildenafil treatment (Figure 1B).

### Plasma angiotensin measurements

**Figure 2:**
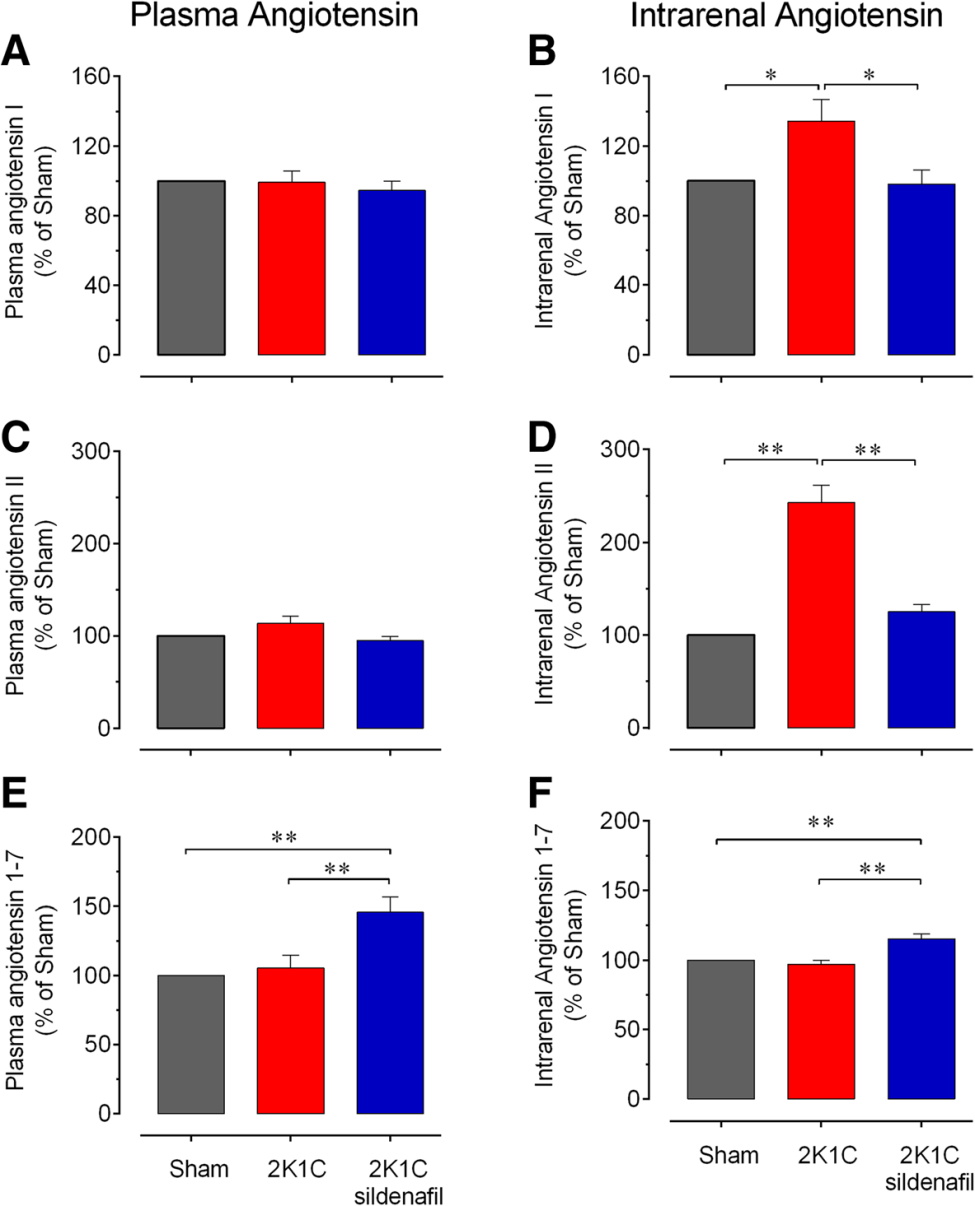
**Effect of sildenafil on angiotensin peptides.** Plasma and intrarenal levels of angiotensin I **(A and B)**, II **(C and D)** and 1–7 **(E and F)** in 2K1C hypertensive mice and 2K1C mice treated with sildenafil compared with the Sham group value (100%). Values are means±SEM for n = 5 to 8 animals per group. *p < 0.05 and **p < 0.01, one-way ANOVA.

Figure 2 shows the average values of the levels of the plasma (bar graphs A, C and E) and intrarenal (bar graphs B, D and F) Ang peptides 28 days after the induction of hypertension by clipping the renal artery. Analysis of the plasma levels of Ang revealed normal levels of Ang I, a tendency of increased levels of Ang II (14%, p > 0.05) and normal levels of Ang 1–7 in the hypertensive 2K1C mice compared to the Sham mice (Figure 2A, C and E). The 2K1C mice treated with sildenafil exhibited plasma levels of Ang I and II that were similar to those of the sham mice. However, sildenafil treatment induced a marked increase (46%, p < 0.01) in the plasma levels of Ang 1–7 in the hypertensive 2K1C mice (Figure 2E). The intrarenal levels of Ang I were slightly higher (34%, p < 0.05) and those of Ang II were markedly higher (143%, p < 0.01) in the hypertensive 2K1C mice compared with the Sham mice (Figure 2B and D). However, sildenafil treatment abolished these increases in the intrarenal levels of Ang I and II (Figure 2B and D) and significantly increased the normal levels of intrarenal Ang 1–7 (15%, p < 0.01) (Figure 2F).

### Vascular function

**Figure 3:**
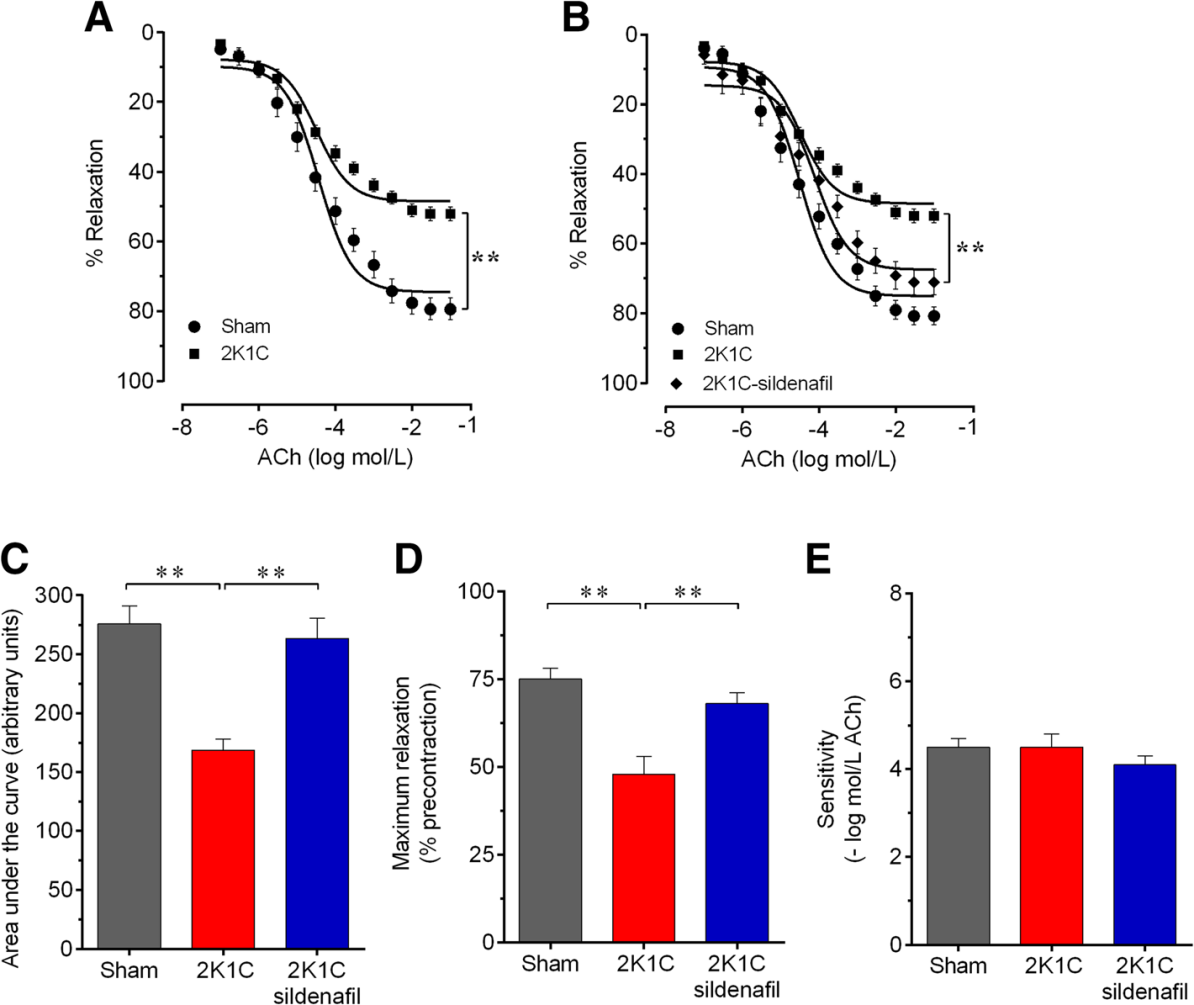
**Effect of sildenafil treatment on the endothelial function.** Acetylcholine-induced relaxation of mesenteric arterial bed preconstricted using norepinephrine, comparing 2K1C mice treated with sildenafil with non-treated 2K1C hypertensive and sham control mice **(A and B)**. Bar graphs show the area under the curve **(C)**, the maximum relaxation (Rmax) **(D)** and the sensitivity (pEC50) **(E)**. Values are the means±SEM for 8–10 animals per group. **p < 0.01, one- **(C, D and E)** and two-way **(A and B)** ANOVA.

The endothelium-dependent relaxation in response to ACh at a constant flow rate in a perfused MAB from each group is shown in Figure 3. The dose–response curves clearly showed a marked impairment of vascular vasodilation in response to ACh in the non-treated hypertensive 2K1C mice compared with the Sham mice (Figure 3A), but this difference was abolished in the 2K1C group chronically treated with sildenafil (Figure 3B). The calculation of the AUCs revealed a significant reduction in the AUC of the hypertensive 2K1C group (~40%, p < 0.01) compared with the sham group. Treatment with sildenafil significantly recovered the AUC to 88% of that of the sham group (Figure 3C). The maximum vascular response (R_max_) was significantly decreased in the MABs from non-treated 2K1C mice (−36%, p < 0.01) but not in those from hypertensive 2K1C mice treated with sildenafil (−11%, p > 0.05), compared to those from the Sham mice (mean of 75±3%, p < 0.01) (Figure 3D). No significant differences were detected in the pEC_50_ between the groups (Figure 3E). In an independent protocol, we examined the functionality of the endothelium-independent vasodilation using NO donor SNP. No significant differences were detected between the three groups of animals (data not shown).

Because endothelial dysfunction in cardiovascular pathologies is typically associated with 1) decreased NO production and/or increased metabolism, 2) decreased prostacyclin (PGI_2_) synthesis, 3) increased production of contractile cyclooxygenase-derived prostanoids, 4) altered endothelium-dependent hyperpolarizing factor (EDHF) levels and 5) increased production of ROS [15,22], we designed additional experiments to examine various molecular pathways.

**Figure 4:**
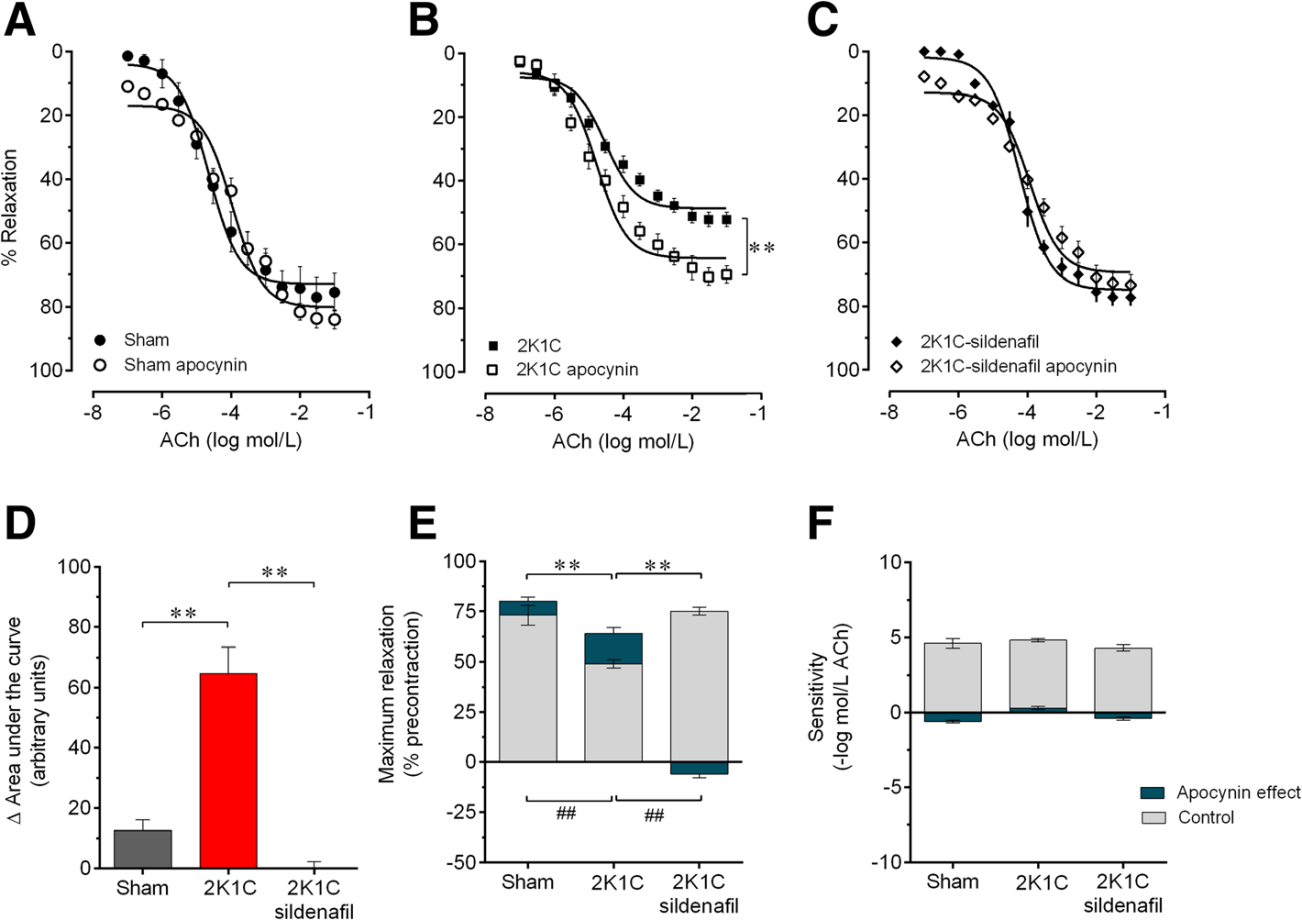
**Contribution of reactive oxygen species to the endothelial dysfunction.** Data show the effect of the blockade of NAD(P)H oxidase activity with apocynin on the dose–response curve to acetylcholine (ACh) in Sham **(A)**, non-treated 2K1C **(B)** and 2K1C mice treated with sildenafil **(C)** mice. Bar graphs show the area under the curve **(D)**, maximum response **(E)** and the sensitivity (pEC_50_) (**E** and **F**, gray color) and the relative changes induced by apocynin (**E** and **F**, blue color) in the groups of animals. Values are the means±SEM for 8–10 animals per group. **p < 0.01 (gray bars) and ^##^p < 0.01 (apocynin effect, green bars), one- **(D,** **E and F)** and two-way **(A,** **B and C)** ANOVA.

The participation of ROS in the relaxation response to ACh was assessed via pre-incubation of the MABs in apocynin, an inhibitor of NADPH. Figure 4 summarizes the dose–response curves in response to ACh for the three groups. As expected, in sham mice (Figure 4A and D), apocynin blockade did not induce a significant change in the ΔAUC (+13 a.u., p > 0.05). In contrast, the hypertensive 2K1C mice exhibited an improved vasodilation response to ACh (Figure 4B), as indicated by an increase in the ΔAUC (+65 a.u., p < 0.01, Figure 4D). In the hypertensive 2K1C mice treated with sildenafil, in which the dose–response curve in response to ACh in the absence of apocynin treatment was similar to that of the Sham mice (Figure 4C), apocynin treatment did not induce a significant change in the ΔAUC (Figure 4D). Similarly, preincubation of the MABs with apocynin did not induce a significant change in the R_max_ to ACh in Sham mice (from 73±5% to 80±2%, Δ + 10%, p > 0.05) or in 2K1C mice treated with sildenafil (from 75±2% to 69±3%, Δ-8%, p > 0.05). In contrast, in 2K1C hypertensive mice, apocynin treatment induced an improvement in the R_max_ (from 49±2% to 64±3%, Δ + 30%, p < 0.01) (Figure 4E). However, there was no significant difference in the sensitivity (pEC_50_) between the three groups of animals (Figure 4F). These data indicate that increased ROS production significantly contributes to the impairment of endothelium-dependent relaxation in MABs from hypertensive 2K1C mice and that sildenafil treatment restores the normal oxidative balance in these animals.

**Figure 5:**
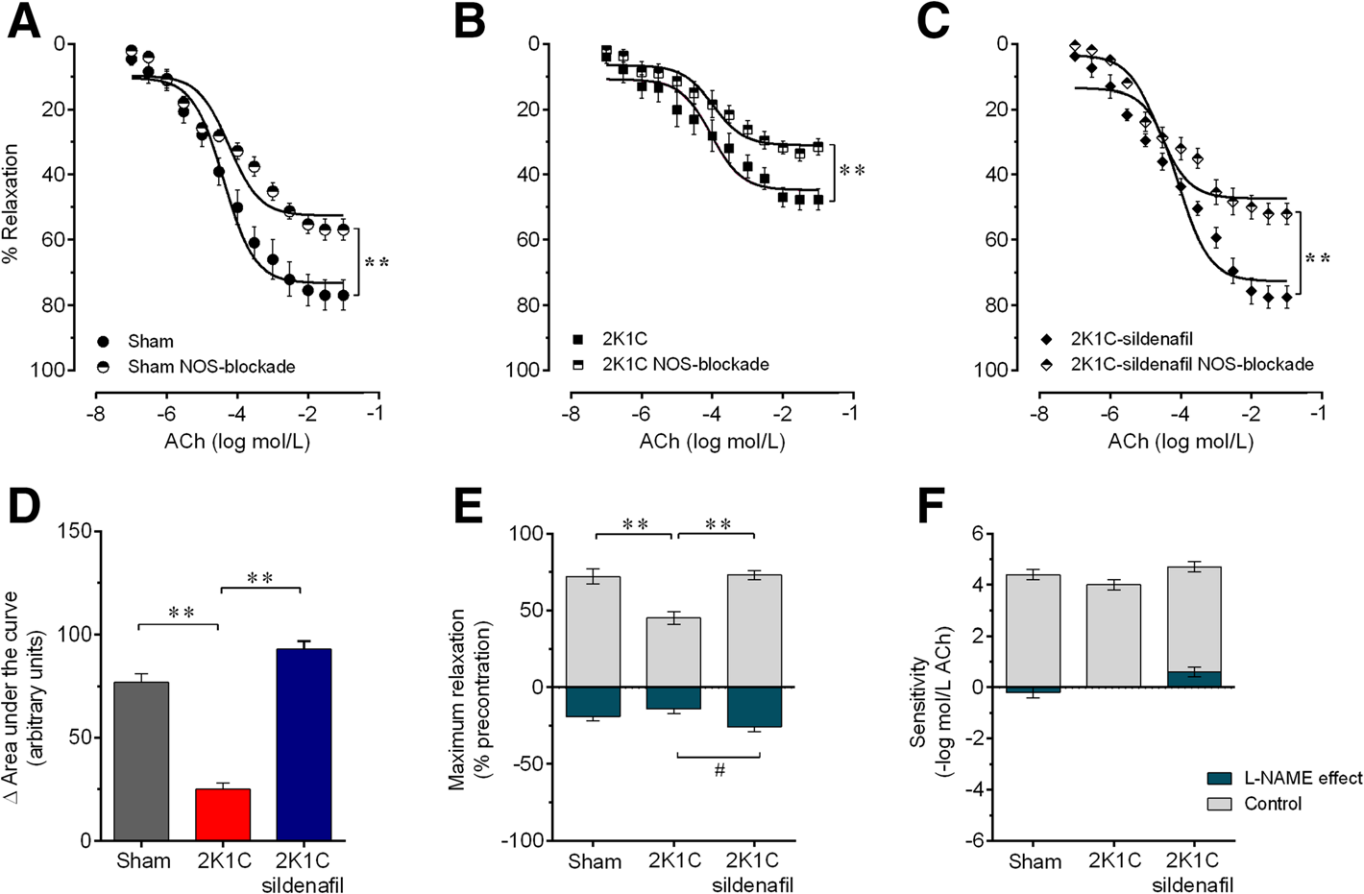
**Contribution of nitric oxide to the endothelial function.** Dose–response relaxation curves to acetylcholine (ACh) in Sham **(A)**, non-treated 2K1C hypertensive **(B)** and sildenafil-treated 2K1C **(C)** mice with and without blockade of nitric oxide synthase (NOS) with L-NAME. Bar graphs show the area under the curve derived from the dose–response curves to ACh **(D)**, the maximum response (R_max_) **(E)** and pEC_50_ **(F)**. Values are the means±SEM. **p < 0.01 and ^#^p < 0.05 compared to the group indicated by the line; two-way ANOVA **(A,** **B and** **C)** and one-way ANOVA **(D,** **E and F)**.

Figure 5 summarizes the data of the vasodilation dose–response curves in response to ACh under the conditions of NOS blockade using L-NAME in MABs from the three groups of animals. Under L-NAME-mediated NOS blockade, the ΔAUC was decreased in Sham mice (−77±5 a.u., Figure 5A and D) and mainly in the hypertensive 2K1C mice (−26±4 a.u., Figure 5B and D), and these alterations were ameliorated by treatment of the 2K1C mice with sildenafil (−93±4 a.u., Figure 5C and E, p < 0.01). The dose–response curves in response to ACh in the presence of L-NAME displayed a significant reduction in the R_max_ in the Sham mice (from 72±5% to 53±3%, Δ-19%, p < 0.01, Figure 5E). In the non-treated 2K1C mice, in which the vasorelaxation response to ACh was already markedly impaired, L-NAME blockade caused a smaller but significant decrease in the perfusion pressure, as R_max_ was further decreased (from 45±4% to 31±2%, Δ-14%, p < 0.01, Figure 5E). In the 2K1C mice treated with sildenafil, the dose–response curves in response to ACh in the presence of L-NAME displayed a similar alteration in the perfusion pressure (from 73±3% to 47±4%, Δ-26%, p < 0.01, Figure 5E) to that of the Sham mice. No significant difference in the pEC_50_ was detected between the three groups (Figure 5F). Taken together, these results indicate that treatment of hypertensive 2K1C mice with sildenafil restores the decreased NO bioavailability in the MAB to that of normotensive Sham mice.

**Figure 6:**
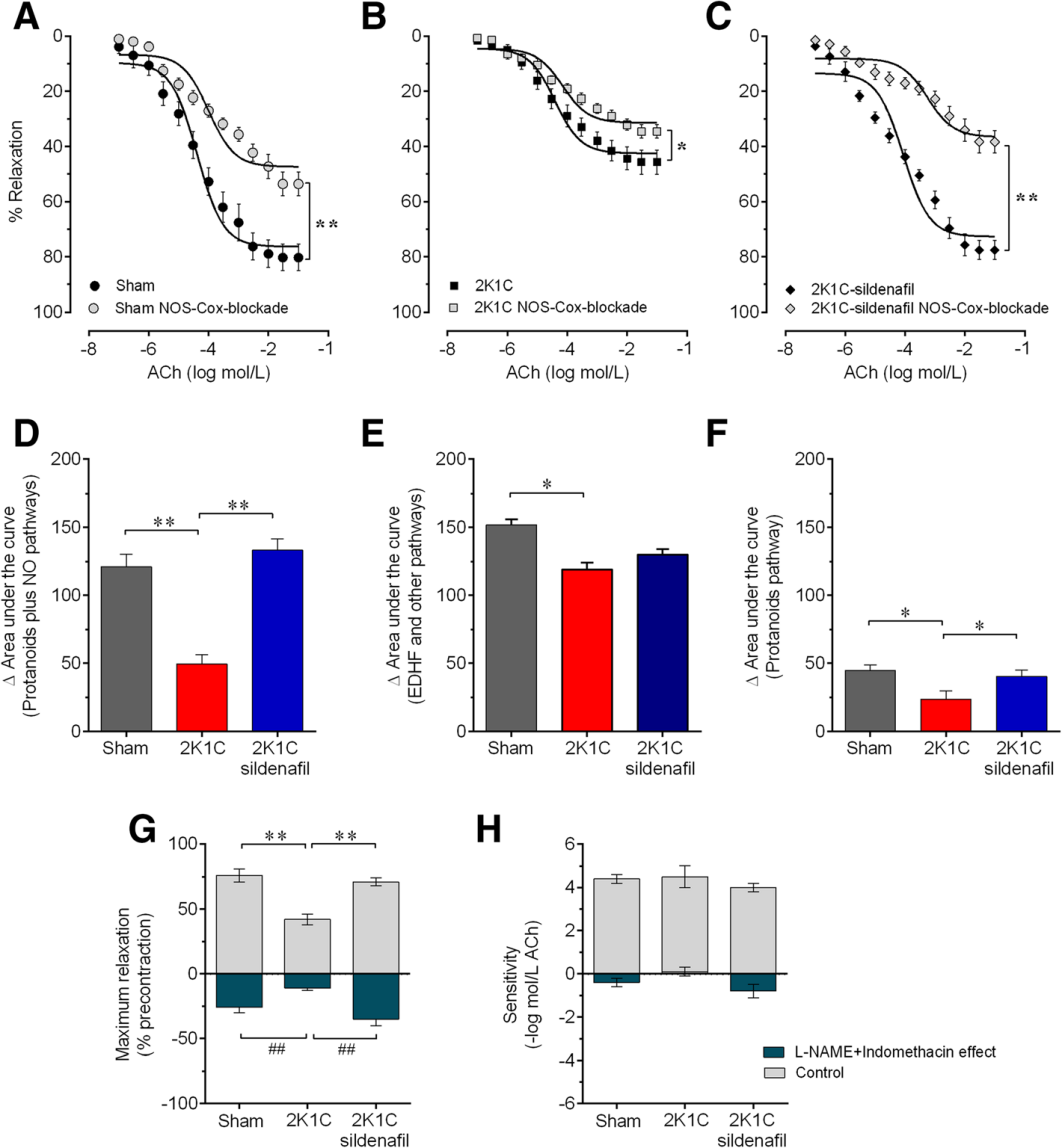
**Contribution of prostanoids and other relaxing pathways to the endothelial dysfunction.** A: dose–response relaxation curves to acetylcholine (ACh) in Sham **(A)**, non-treated 2K1C hypertensive **(B)** and sildenafil-treated 2K1C **(C)** mice. Dose–response curves to ACh were obtained with and without the double blockade of NOS with L-NAME and of cyclooxygenases with indomethacin. Bar graphs show the area under the curve (AUC) after the double blockade **(D)**, the total AUC minus the AUC after the double blockade (contribution of EDHF and other pathways, **E**) and the AUC derived from the AUC under double blockade minus the AUC under the L-NAME blockade (prostanoids pathway, **F**). Graphs **G** and **H** show the effects of indomethacin on the maximum relaxation (R_max_, **G**) and sensitivity (pEC_50_, **H**) and on these two parameters. Values are the means±SEM for 8 to 10 animals per group. *p < 0.05, ^#^p < 0.05, **p < 0.01and ^##^p < 0.01, compared with the group indicated by the line; two-way ANOVA **(A,** **B and C)** and one-way ANOVA **(D-H)**.

To indirectly quantify the contribution of prostanoids and other pathways not involving NO or prostanoids to the relaxation response induced by ACh, we designed an experiment in which two inhibitors, L-NAME and indomethacin, were applied (Figure 6). Thus, the difference in the AUC between treatment with both inhibitors and treatment with L-NAME alone indicates the contribution of the prostanoids pathway (Figure 6F), and difference between the AUC under control conditions and the AUC in the presence of both inhibitors indicates the contribution of EDHF and other unidentified vasodilatory pathways (Figure 6E). The dose–response curves in response to ACh under in the presence of both inhibitors were significantly shifted upward (reduced perfusion pressure, i.e., reduced vasodilation) in all three groups of animals, although to a lesser extent in the hypertensive 2K1C group (Figure 6B) and to a greater extent in the 2K1C group treated with sildenafil (Figure 6C) compared with the Sham group (Figure 6A). The AUC (before - after treatment with both inhibitors), as shown in Figure 6D), was significantly reduced in the Sham mice (−122 a.u., p < 0.01) and was markedly increased in the hypertensive 2K1C mice (50%, p < 0.01), but recovered by treatment with sildenafil (−133 a.u., p < 0.01). The contribution of prostanoids to the change in the AUC was significantly reduced in the hypertensive 2K1C mice (47%, p < 0.01) compared with the Sham mice (45±4 a.u.); sildenafil treatment restored these values to control levels (40±5 a.u.) (Figure 6F). The contribution of EDHF and other unidentified pathways was greater than that of the prostanoids pathways, as indicated by the average values shown in Figure 6E. The ΔAUC associated with EDHF and other unidentified vasodilation pathways was significantly reduced in the hypertensive 2K1C mice (30%, p < 0.01) compared with the Sham mice (Figure 6E). This difference was abolished by treatment with sildenafil. The R_max_ in the presence of both inhibitors was significantly decreased in all three groups (Figure 6G), although to a lesser extent in the hypertensive 2K1C group (from 42±4% to 31±2.0%, Δ-11%, p < 0.01) and to a greater extent in the 2K1C group treated with sildenafil (from 71±3% to 36±5%, Δ-35%, p < 0.01) compared with the Sham group (from 76±5% to 48±4%, Δ-38%, p < 0.01). The sensitivity (pEC_50_) was similar between the three groups both before and after treatment with both inhibitors (between groups), but in the 2K1C mice treated with sildenafil, treatment with both inhibitors induced a significant decrease (−20%, p < 0.05, Figure 6H).

**Figure 7:**
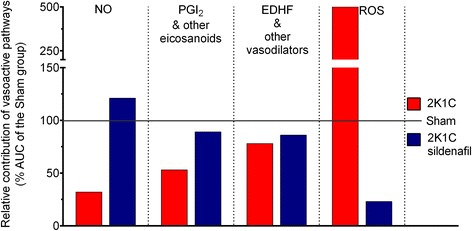
**Schematic diagram illustrating the individual contribution of each endothelial pathway to the endothelial function in the RAS-dependent renovascular hypertension.** Data show percentage of changes in the area under the curve (AUC) in relation to the Sham group (100%) and was derived from the difference in the area under the dose–response curve to acetylcholine (Figures 3, 4, 5 and 6) before and after the blockade of the molecular pathways; endothelial nitric oxide (NO) synthase (with L-NAME), cyclooxygenases (Cox)/prostanoids such as prostacyclin (PGI_2_) and other eicosanoids (with indomethacin), reactive oxygen species (ROS)/NADPH oxidase (with apocynin) and endothelial-derived hyperpolarizing factor (EDHF) (residual response after double blockade with L-NAME and indomethacin).

The bar graph in Figure 7, which was generated using the AUCs from dose–response curves to ACh from Figures 3, 4, 5 and 6, displays the relative contribution of each molecular pathway to vasodilatory process (difference between the total AUC and the AUC after treatment with both L-NAME and indomethacin) and the opposing effect of the ROS pathway. The total AUC of the Sham group (275±13 a.u.) was considered as 100% and was used as the reference value for comparisons with the non-treated 2K1C group (169±11 a.u.) and the 2K1C group treated with sildenafil (263±15 a.u.). As shown, the contribution of the NO/cGMP pathway was markedly impaired in the hypertensive 2K1C mice (34%), which were recovered by sildenafil treatment (121%). The prostanoids/cAMP pathway, which includes PGI_2_ and other vasodilatory eicosanoids, was also substantially impaired in the hypertensive 2K1C mice (53%), and sildenafil treatment attenuated this impairment (89%). The residual area of the total AUC after treatment with both inhibitors (L-NAME and indomethacin) corresponds to the contribution of other known vasodilatory factors, such as EDHF/K^+^ channels, and, in our opinion, it could also include the Ang 1–7; this pathway was slightly attenuated (78%) and was incompletely restored by sildenafil treatment (86%). The ROS levels were markedly increased in the hypertensive 2K1C mice (500%), and we found that its inhibitory effect on the vasodilation process was alleviated by sildenafil treatment (23%).

### Flow cytometry analysis of oxidative stress in endothelial cells from MAB

**Figure 8:**
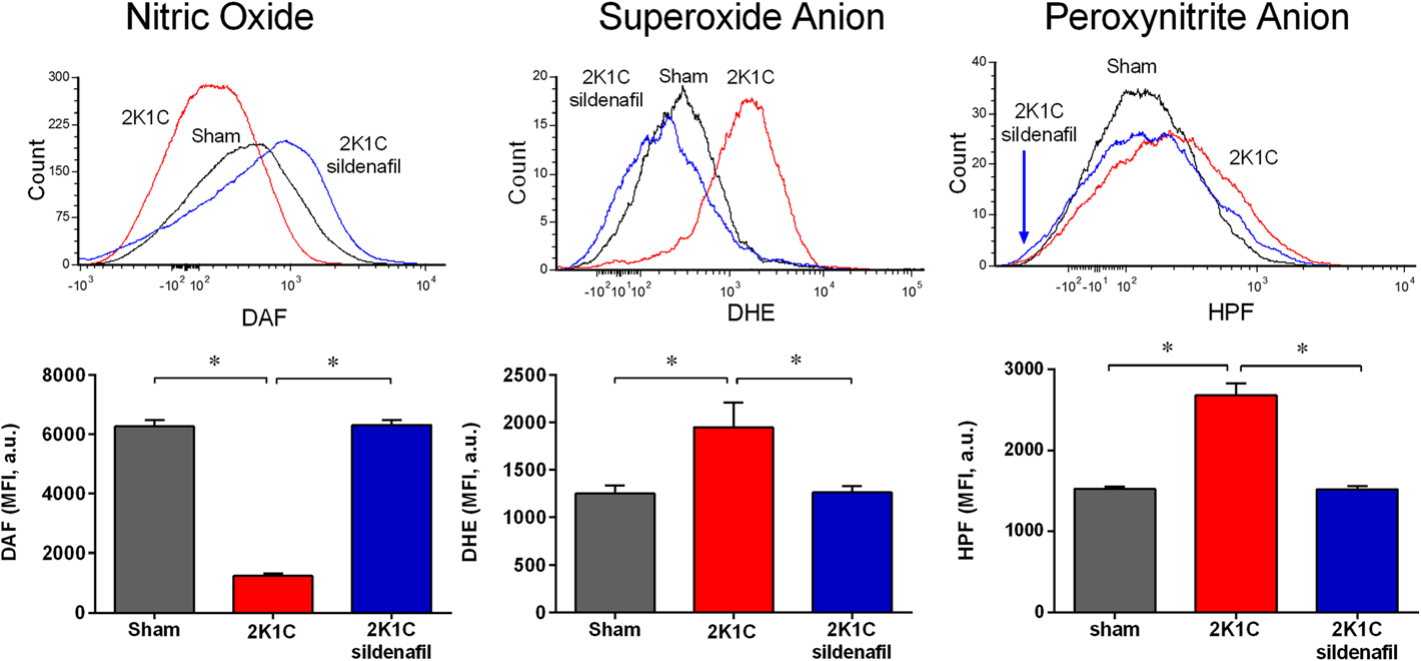
**Superoxide anion production in mesenteric vascular beds.** Top panel shows representative histograms from flow cytometric analysis using dihydroethidium (DHE), diaminofluorescein (DAF) and hydroxyphenyl fluorescein (HPF) in Sham, 2K1C hypertensive and sildenafil-treated 2K1C mice; the log fluorescence (X axis) illustrates the differences in intensity of fluorescence for the number of cells counted. Bar graph shows augmented production of superoxide anions and peroxynitrite in 2K1C hypertensive mice compared with Sham mice and sildenafil treatment restored the normal values. On the other hand, 2K1C mice presented impairment of nitric oxide production compared with sham mice, and sildenafil treatment restored the normal levels. Values are median fluorescence intensity (MFI, in arbitrary units) ± SEM for 5 animals per group. *p < 0.05 (one-way ANOVA).

Intracellular •O_2_^−^, •ONOO^−^ and NO in endothelial cells were quantified by flow cytometry through DHE, HPF and DAF staining. Top panels of Figure 8 are typical histograms showing a rightward-shift in the log of DHE and HPF fluorescence in 2K1C hypertensive mice compared with Sham mice and contrasting with a leftward-shift observed in 2K1C animals treated with sildenafil. On the other hand, the typical histogram of NO production of the 2K1C mouse treated with sildenafil, showed a rightward-shift in the log of DAF fluorescence when compared with the 2K1C hypertensive mouse. As summarized in the bar graphs (Figure 8), endothelial cells isolated from MAB of 2K1C mice exhibited a remarkable increase in •O_2_^−^ and •ONOO^−^ levels (41% and 76%, respectively, p < 0.05) when compared with the levels of Sham mice (1252±81 and 1524±25 a.u., respectively). However, the chronic treatment of 2K1C mice with sildenafil was able to decrease the production of •O_2_^−^ and •ONOO^−^ to levels (1266±25 and 1518±40 a.u., respectively, p < 0.05) similar to that observed in the Sham group. Additionally, sildenafil treatment of 2K1C mice restored the NO levels (6310±172 a.u., p < 0.05) when compared with Sham (6273±218 a.u.) and the non-treated 2K1C mice (1242±25 a.u.).

## Discussion

Previous results from our laboratory [21] demonstrated by the first time that sildenafil repaired the hypoperfused kidney injury in 2K1C hypertensive mice by activation of the NO-mediated pathway. This pleiotropic protective role of sildenafil led us to investigate other additional beneficial effects of this drug on the systemic RAS and resistance vasculature in this model of hypertension. Our primary finding was that chronic treatment of these animals with this selective PDE5 inhibitor sildenafil restored the levels of BP and vascular relaxation to those of normotensive sham mice. Moreover, the present study is the first to reveal that the beneficial effects of sildenafil appear to be mediated by decreasing the levels of the vasoconstrictive and pro-oxidant peptide Ang II and increasing the levels of its physiological antagonist Ang 1–7.

A recent work from Navar’s group and our collaborator Casarini’s group [34] revealed that the maintenance of high blood pressure in 2K1C is dependent of an imbalance between high levels of ACE/Ang II and low levels of ACE2/Ang 1–7, both being influenced by renin activity [16,35]. In parallel, it has been reported that augmentation of chymase activity in the ischemic kidney of 2K1C rats [36] may be a mechanism related with ACE-independent intrarenal Ang II production. Moreover, previous studies have demonstrated that Ang II downregulates ACE2 mRNA expression in some tissues, including the kidney [37,38], which could be mediated by AT1 receptors [39,40]. Based on the above findings and on our studies in 2K1C hypertensive mice, we speculate that the repair of the stenotic kidney by sildenafil (administered for 14 days) was mediated by attenuation of renal baroreceptor activity, with consequent deactivation of the intrarenal RAS, as confirmed in the present study by decrease in intrarenal Ang I (~33%) and II (~50%) and (contrasting) by increase in Ang 1–7 (~15%), as compared with 2K1C mice.

Regarding the plasma Ang I and II, our finding of normalized levels at the chronic phase was expected based on the fact that in the rat Ang-dependent renovascular hypertension has been separated in two phases [5]. The initial phase is related to the development of hypertension, which occurs from the first day following the placement of the clip to the 14^th^ day, when the plasma levels of Ang II peak. The late (second) phase is related to the maintenance of hypertension, which has been evaluated at approximately 28 days after renal artery clipping, and that shows stabilized plasma levels of Ang II [5]. In the mouse model, the few available studies have reported an increase in renin activity and the levels of Ang I (~50%) [20,41], Ang II (~350%) [20,30,42] and Ang 1–7 in plasma (~110%) [20] during the first phase of renovascular hypertension (14 days following the clip placement). During the second phase of 2K1C hypertensive mice, neither increased plasma renin activity nor elevated levels of Ang I [41] or Ang II [42,43] has been detected, justifying the similar profile founded in this study. Altogether, the above data extend to the mice the time-course profile of Ang that has been demonstrated by Navar’s research group in the rat [5]. Surprisingly, we observed that sildenafil treatment increased plasma levels of Ang 1–7 (~45%), which is known to counterbalance the deleterious effects elicited by Ang II [16,35]. Based on our findings, we speculate that the Ang 1–7 generated by the ACE2-mediated degradation of Ang II may also limit the intrarenal uptake of Ang II, as discussed above. It should be noted that, in a review of contemporary literature, it is notable a vast diversity in the methods that have been used for the analysis of the RAS in the few studies using the 2K1C mouse model, confounding the comparison of the plasma and tissue levels of RAS peptides. As a solution for this discrepancy, we considered that it would be most appropriate to compare our detected values in the hypertensive 2K1C mice and the 2K1C mice treated with sildenafil to the average values in the Sham mice, which were normalized to 100%; similarly, we will compare the data found by us and others using relative (%) values.

In the present study, 2K1C mice exhibited a high BP, approximately 20% higher than that of the Sham mice, in agreement with other studies reporting similar increases (20-25%) [12,15,30,31,41-45]. The high BP was accompanied by tachycardia, which is consistent with other reports [20,21,41]. As expected, and based on previous studies using the rat model [21,46-48], we found that sildenafil normalized the BP and HR. Moreover, the novelty of the present study is that according to the profile of intrarenal and plasma Ang that we observed, it could be speculated that these beneficial effects of sildenafil are partially mediated by the reciprocal changes in Ang, i.e., a decrease of Ang II and increase of Ang 1–7. This hypothesis may be reinforced by the finding that chronic treatment of spontaneously hypertensive rats (SHR) with Mas receptor agonists or with Ang 1–7 led to vasorelaxation, anti-hypertensive and cardioprotective effects [49,50]. Additionally, the plasma levels of Ang 1-7 were increased during treatment with ACE inhibitors or AT1 receptor blockers, indicating a potential dependence of the anti-hypertensive effects of those compounds on Ang 1–7 [18,39]. Despite of our results demonstrate a drop in Ang II, we cannot rule out the possibility the participation of AT2 receptor in this model of hypertension, since this protein can stimulates NO signaling [51], potentiating the effects of sildenafil. This hypothesis is corroborated by other recent studies that demonstrated the importance of AT2 receptor in renovascular hypertension [52,53]. Thus, targeting the ACE2-Ang (1–7)-Mas and AT2 receptor axis using new pharmacological tools, such as sildenafil, which we used in the current study, may represent a novel therapeutic strategy for renovascular hypertension.

It has been demonstrated that the interruption of the ACE2-Ang (1–7)-Mas axis is an underlying mechanism responsible for the worsening of the developmental and maintenance phases in 2K1C Mas knockout mice [39], as well as impairing the baroreflex control, endothelial function and the balance of NO/ROS in normotensive mice [40,41]. In parallel, recent data have demonstrated specific and beneficial effects of AT2 receptor via central areas in hypertensive rats [54]. Considering the evidence that sildenafil crosses the blood–brain barrier [55,56] and that it affects the angiotensin profile, one cannot rule out the possibility of also a central action on the sympathetic/parasympathetic drive. In this context, although recent studies have shown an increased sympathetic drive imposed on the cardiovascular system by sildenafil in both rats [56] and humans [57,58], we speculate that in our study (in which sildenafil was chronically administered) its systemic antioxidant effects overtook the possible undesirable sympathetic-mediated effects.

Endothelial dysfunction is an important abnormal function commonly observed in cardiovascular diseases, including atherosclerosis [22] and various models of arterial hypertension [15]. Previous studies by our group revealed that the MAB exhibits an increased response to vasoconstrictors and a decreased response to vasodilators in RAS-dependent hypertensive rats [24,59] and mice [15]. Thus, in the current study, we designed an experiment to evaluate the effects of sildenafil treatment on endothelial dysfunction in hypertensive 2K1C mice. We selected the 2K1C model that was developed by Goldblatt decades ago because endothelial dysfunction is an early characteristic of RAS-dependent 2K1C-mediated hypertension [15,60]. As expected, ACh evoked endothelium-dependent relaxation, which was greatly diminished in the 2K1C mice. However, no significant difference was detected in the dose–response curve to the NO donor SNP, indicating that vascular dysfunction may be due to dysfunctional molecular pathways in endothelial cells but not in vascular smooth muscle cells. This result is in agreement with studies showing that impaired endothelium-dependent vasorelaxation is associated with the second phase of chronic 2K1C hypertension in the rat model [5,61]. Because we hypothesized that 2K1C-induced endothelial dysfunction may be improved by chronic treatment with sildenafil, standard experiments using pharmacological inhibitors were designed to assess the relative contribution of each molecular pathway to the relaxation responses to ACh in the MAB and the effectiveness of sildenafil in restoring or improving endothelial function in the 2K1C mice.

The relative importance of vasoactive substances, such as NO, PGI_2_, EDHF and ROS, depends on the size of the blood vessels and the experimental animal. Studies have shown that in the rat, NO is the primary contributor to ACh-induced vasodilation in the aorta, whereas EDHF plays a predominant role in resistance vessels [62]. Our data show that in both the wild type and hypertensive 2K1C mice, the EDHF pathway was the primary contributor and was scarcely altered in the endothelium-dependent relaxation response to ACh in the MAB. This finding corroborates a previous study showing that EDHF-mediated dilatory responses were unaffected in mesenteric resistance vessels in RAS-dependent hypertensive mice [63]. Moreover, our data show that in the vasodilator pathway, the impairment of NO/cGMP was the primary contributor to endothelial dysfunction, followed by PGI_2_, as determined using hypertensive 2K1C mice. Indeed, the contribution of NO and PGI_2_ to endothelial function in the 2K1C mice was reduced to approximately 35% and 50%, respectively, of that in the Sham mice. By blocking NAD(P)H using apocynin, we also found that the levels of ROS, which have opposing effects to those of NO and PGI_2_, were increased approximately 6-fold in the 2K1C mice, in agreement with the rat model of renovascular hypertension [48,64]. This finding may be because high BP (enhanced shear stress) and activation of the RAS have been shown to increase ROS production by NAD(P)H oxidase via the AT_1_ receptor axis, decreasing NO bioavailability in the 2K1C mice [27,30,45,65]. The hypothesis that it occurs an imbalance between NO and O_2_ levels in the endothelial cells isolated from MAB, but not only systemically [21,30,31], was confirmed in a separated set of experiments by flow cytometry in the present study. Additionally, by using the HPF staining, we found in renovascular hypertensive mice an increase of endothelial production of peroxynitrite, which is derived from the reaction of NO with the superoxide anion. Taken together, these results suggest a relevant role of impairments of the NOS/cGMP and prostanoid/cAMP pathways and the activation of the ROS/cGMP pathway in the endothelial dysfunction observed in RAS-dependent hypertension [66].

The second novel finding based on endothelial function analysis is that sildenafil recovered endothelial function to normal levels, as indicated by the parameters of the dose–response curve to ACh (R_max_ and AUC) and flow cytometry analysis of ROS. The recovery of endothelial function by sildenafil treatment primarily occurred due to a marked increase in the vasodilatory contribution of NO and a marked decrease in the levels of superoxide and (consequently) peroxynitrite anions. Recent data have shown that this vasodilator effect of sildenafil may involve indirect mechanisms, such as upregulation of BH_4_ and soluble guanylate cyclase (sGC) enzyme, besides the inhibition of NADPH oxidase activity, leading to the increase of NO bioavailability [67]. These findings corroborate the notion that the inhibition of PDE5 in the vessels in smooth muscle directly enhances the NO/cGMP signaling pathway by inhibiting cGMP degradation and subsequently facilitating vessel relaxation [68].

## Conclusion

In conclusion, our data show that the chronic treatment of Ang-induced renovascular hypertensive mice with the PDE5 inhibitor sildenafil leads to a decrease in high BP and tachycardia, probably by decreasing the levels of plasma and intrarenal Ang II and increasing the levels of its physiological antagonist Ang 1–7. Additionally, sildenafil was able to abolish the endothelial dysfunction and the increased production of •O_2_^−^ of resistance vessels and then restoring the NO/ROS balance. The above data and interpretation indicate that the activation of the ACE2-Ang (1–7)-Mas axis by sildenafil may represent a novel therapeutic target in the context of endothelial dysfunction. Therefore, the present data revealing the beneficial effects of sildenafil support the concept that this inhibitor of PDE5 could be included in the hallmark of anti-hypertensive agents currently available, especially as an additional drug in patients with resistant hypertension despite the use of three or more drugs and in patients with endothelial dysfunction and kidney disease.

### Limitations and perspectives

Although the present study was designed to assess the functional beneficial effects of sildenafil, a limitation is that our experiments did not completely elucidate the mechanisms underlying the specific molecular pathways by which sildenafil exerts its beneficial effects; we plan to address this issue in further studies. However, this translational research is of clinical relevance because it reveals new concept of interaction of sildenafil with the RAS and opens the perspective of using sildenafil to stimulate the production of Ang 1–7 as a new therapeutic approach for the treatment of renovascular hypertension an endothelial dysfunction, as well as for resistant hypertension.

## Abbreviations

2K1C: Two-kidney, one-clip
ACh: Acetylcholine
ACE: Angiotensin converting enzyme
Ang II: Angiotensin
BP: Blood pressure
DHE: Dihydroethidium
EDHF: Endothelium-derived hyperpolarizing factor
eNOS: Endothelial nitric oxide synthase
HR: Heart rate
L-NAME: N-nitro-L-arginine methyl ester
MAB: Mesenteric arterial bed
MAP: Mean arterial blood pressure
NAD(P)H: Nicotinamide adenine dinucleotide phosphate
NO: Nitric oxide
PDE: Phosphodiesterase
RAS: Renin-angiotensin system
ROS: Reactive oxygen species
SNP: Sodium nitroprusside
